# Fetal Outcomes in Preterm Cesarean Sections

**DOI:** 10.7759/cureus.27607

**Published:** 2022-08-02

**Authors:** Sundus Rahman, Mohib Ullah, Asma Ali, Nighat Afridi, Humaira Bashir, Zahra Amjad, Aliya Jafri, Areeba Jawaid

**Affiliations:** 1 Department of Obstetrics and Gynecology, Combined Military Hospital (CMH), Peshawar, PAK; 2 Department of Medicine, Islamabad International Hospital and Research Centre, Islamabad, PAK; 3 Department of Obstetrics and Gynecology, Ayub Teaching Hospital, Abbottabad, PAK; 4 Department of Obstetrics and Gynecology, Mufti Mahmood Memorial Teaching Hospital, Dera Ismail Khan, PAK; 5 Department of Medicine, Geo Life Hospital, Dera Ismail Khan, PAK; 6 Department of Biochemistry, Jinnah Sindh Medical University, Karachi, PAK; 7 Department of Internal Medicine, King's College Hospital, London, GBR

**Keywords:** spontaneous vaginal delivery, neonatal mortality, small for gestational age, preterm, infant, cesarean section, apgar

## Abstract

Introduction

Neonatal mortality is a major challenge in low-middle-income countries. The current study was conducted to assess the association between preterm cesarean delivery and fetal outcomes.

Methods

A prospective study was conducted at the Combined Military Hospital in Peshawar, Pakistan, from October 1, 2020, to March 31, 2021. All women reporting to the hospital with a cephalic presentation and singleton pregnancies between the 27th and 34th weeks of gestation were included in the study. Pregnancies with an abnormal presentation, those diagnosed with a congenital abnormality, and those with indications for growth restriction or preterm delivery were excluded from the study. We also excluded infants delivered via vacuum or forceps. The outcomes of interest in this study included neonatal death prior to discharge, neonatal respiratory distress, sepsis, intraventricular hemorrhage (IVH), seizure, subdural hemorrhage (SDH), or appearance, pulse, grimace, activity, and respiration (APGAR) test score of less than 7 at five minutes. Maternal features including diabetes, hypertension and gestational age of delivery, parity, previous cesarean sections (CS), and last pregnancy outcomes were documented in a predefined pro forma.

Results

Our sample size consisted of 288 women, who were classified into two groups. Group A comprised 144 women who gave birth vaginally and group B consisted of 144 women who underwent CS. It was observed that women who underwent cesareans had a higher likelihood of a history of hypertension and related pathologies. It was also observed that these women had a greater likelihood of being of higher age compared to women who underwent vaginal deliveries. Neonates of women who had CS were at a greater risk of presenting with respiratory distress than those who had spontaneous vaginal deliveries.

Conclusion

Based on our findings, respiratory distress was significantly more common in babies of women who delivered via CS. However, there was no difference in neonatal outcomes in terms of IVH, seizures, SDH, and APGAR score of <7.

## Introduction

Cesarean section (CS) is significantly more common in the birth of preterm neonates than full-term neonates [1]. Non-reassuring fetal status in premature pregnancies is the most common indication for performing a CS. Nevertheless, any clear association between fetal outcome and CS in preterm delivery has yet to be established. CS constitutes a significant economic burden and is associated with a high maternal morbidity rate. These facts should be acknowledged when opting for CS as the preferred route of delivery and thus this intervention should be reserved for cases where it can confer an unambiguous advantage for either the mother or the child [2-4].

Neonates weighing <1500 grams, i.e., very preterm or very-low-birth-weight (VLBW) babies, have been studied in terms of health outcomes after CS and vaginal delivery. The literature provides contradictory conclusions with regard to neonatal outcomes such as the incidence of intraventricular hemorrhage (IVH) and mortality [5]. Deulofeut et al. studied the outcomes of vaginal and CS delivery in VLBW and extremely-low-birth-weight (ELBW; birth weight <1000 grams) neonates weighing <1252 grams. They reported that vaginal delivery is not associated with negative outcomes in the short term for neonates in their entire cohort but is consistently associated with short-term negative outcomes and IVH in neonates weighing ≤751 grams at birth. They also concluded that the incidence of poor outcomes is lower in neonates who weigh more at birth [6,7]. Lee et al. suggested that for low-birth-weight (LBW) neonates who are not small for gestational age (SGA), vaginal delivery reduces mortality rates [8]. However, many studies have demonstrated no significant benefit or risk of one route of delivery over the other for premature neonates who are not SGA [9].

Mothers who give birth via CS are at increased risk of developing short- and long-term risks; these include excessive bleeding, formation of adhesions, increased risk of negative surgical outcomes in the future, and postoperative infections [10]. CS also leads to greater economic losses in contrast to vaginal deliveries and thus adds to the economic burden [11]. Thus, like all surgical interventions, CS needs to be justified by showing clear benefits to maternal and neonatal outcomes in relation to the costs of procedure and post-procedure care. Werner et al. conducted a study on preterm but SGA neonates, demonstrating that CS did not confer a statistically significant advantage over vaginal deliveries [12]. In light of the conflicting evidence in the literature, our study aims to evaluate the neonatal outcomes of CS in neonates born between 24-34 weeks of gestation.

## Materials and methods

Study design and setting

A prospective study was conducted at the Combined Military Hospital, Peshawar from October 1, 2020, to March 31, 2021. After obtaining ethical clearance (Reference # CMH/GEN/887-7) from the Institutional Review Board at the Combined Military Hospital and informed consent from the patients, the phase of data acquisition was initiated.

Patient selection

Participants were recruited using non-probability consecutive sampling. The sample size was determined by using select statistics software by keeping the rate of respiratory distress as 25.6% and 39.2% in neonates born vaginally and via CS, respectively. The confidence level was kept at 90% and power was taken as 80% to yield a sample size of 288 (144 in each group).

The inclusion criteria were as follows: pregnant women carrying singleton fetuses at 24 total weeks to 34 total weeks of gestation with a cephalic presentation. The exclusion criteria were as follows: neonates born via operative delivery (e.g., forceps or vacuum) as this is an independent risk factor for death and disability in preterm neonates, fetuses with congenital abnormalities, fetal presentations other than cephalic, and evidence of growth restriction or SGA.

Data collection

Informed verbal and written consent was obtained from all pregnant women after informing them about the significance of the study. A predefined proforma was used to collect data on neonatal outcomes of women who delivered via CS and those who had a vaginal delivery. The neonatal outcomes documented were as follows: appearance, pulse, grimace, activity, and respiration (APGAR) test score at five minutes, mortality before and at discharge, respiratory distress, IVH, subdural hemorrhage (SDH), the incidence of fits/seizures, and systemic infections (sepsis). Based on the APGAR score, two groups of neonates were formed: those with a score >7 and those with a score <7. Maternal features including a past medical history of hypertension and diabetes mellitus, weeks of gestation at delivery, parity, previous CS, and last pregnancy outcomes were recorded in a predefined pro forma.

Statistical analysis

Data analysis was carried out using SPSS Statistics version 26 (IBM Corp., Armonk, NY). In order to evaluate and analyze associations between the mode of delivery (MOD) and neonatal outcomes, chi-square tests were utilized, and a p-value <0.05 was considered statistically significant.

## Results

Our sample size comprised 288 women. Group A consisted of 144 women who gave birth vaginally and group B consisted of 144 women who underwent CS. Table 1 presents a tabular comparison of maternal features of groups A and B. It was observed that women who underwent CS had a greater likelihood of a history of hypertension and related pathologies. It was also observed that these women had a greater likelihood of being of higher age compared to women who underwent vaginal deliveries.

**Table 1:** Maternal characteristics in vaginal delivery versus cesarean delivery

Maternal characteristics	Vaginal delivery, n (%)	Cesarean delivery, n (%)	P-value
Maternal age, years			0.017
Younger than 20	18 (12.5%)	8 (5.6%)	
20–34	99 (68.7%)	92 (63.9%)	
35 and older	27 (18.8%)	44 (30.6%)	
Parity			0.805
Nulliparous	67 (46.5%)	60 (41.7%)	
One	39 (27.1%)	39 (27.1%)	
Two	19 (13.2%)	23 (16%)	
Three or more	19 (13.2%)	22 (15.3%)	
Maternal education			0.113
High school or less	97 (67.4%)	84 (58.3%)	
More than high school	47 (32.6%)	60 (41.7%)	
Gestational age at delivery, weeks			0.304
Less than 26	8 (5.6%)	6 (4.2%)	
26–28	9 (6.3%)	13 (9%)	
28–30	12 (8.3%)	20 (13.9%)	
30–32	21 (14.6%)	26 (18.1%)	
Greater than 32	94 (65.3%)	79 (54.9%)	
Diabetes	10 (6.9%)	14 (9.7%)	0.394
Hypertensive disorders	14 (9.7%)	53 (36.8%)	<0.001
Smoking	13 (9%)	9 (6.3%)	0.375

Table 2 presents the comparative statistics regarding neonatal outcomes in terms of inpatient death, neonatal respiratory distress syndrome (NRDS), fits/seizures, sepsis, and APGAR score at five minutes. It was observed that neonates of women in group B were at greater risk of presenting with NRDS, having an APGAR score at five minutes of <7, fits, sepsis, and higher mortality.

**Table 2:** Neonatal outcomes in patients with vaginal versus cesarean delivery APGAR: appearance, pulse, grimace, activity, and respiration

Neonatal outcomes	Vaginal delivery, n (%)	Cesarean delivery, n (%)	P-value
Respiratory distress			0.017
Yes	37 (25.7%)	56 (38.9%)	
No	107 (74.3%)	88 (61.1%)	
Sepsis			0.520
Yes	4 (2.8%)	6 (4.2%)	
No	140 (97.2%)	138 (95.8%)	
Intraventricular hemorrhage			0.791
Yes	7 (4.9%)	8 (5.6%)	
No	137 (95.14%)	136 (94.44%)	
Seizures			0.562
Yes	1 (0.69%)	2 (1.39%)	
No	143 (99.31%)	142 (98.61%)	
Subdural hemorrhage			1
Yes	1 (0.69%)	1 (0.69%)	
No	143 (99.31%)	143 (99.31%)	
Five-minute APGAR score of less than 7			0.128
Yes	8 (5.56%)	15 (10.42%)	
No	136 (94.44%)	129 (89.58%)	
Death			0.735
Yes	4 (2.78%)	5 (3.47%)	
No	140 (97.22%)	139 (96.53%)	

## Discussion

The number of cesarean deliveries for early preterm deliveries has remained mostly steady with a minor decline since 2009, while a yearly rise had been observed a decade before 2009. CS is widely carried out for the delivery of neonates at gestational age <34 weeks (i.e. early preterm). According to the Centers for Disease Control (CDC)’s National Center for Health Statistics (NCHS), the rate of CS for early preterm neonates is 32.3% as of 2011 [13]. Various sources in the literature document a correlation between CS and the development of respiratory distress in newborns [14-16]. In contrast, studies have also documented no statistically significant association between CS and the incidence of respiratory distress [9].

The present study engages in a comparison of neonatal outcomes after CS and normal vaginal delivery (NVD). We found that infants born via CS were more likely to develop pulmonary distress. Thus it may be inferred that in light of the adverse outcomes of CS for both the mother and fetus, NVD may be deemed safe, if not advantageous, for the delivery of early preterm neonates.

A study by Benzouina et al. compared elective and emergency CS with regard to indications for the decision to opt for a CS and fetal outcomes. In this study, statistically significant indications for CS were as follows: referrals from peripheral healthcare facilities due to adverse antepartum and intrapartum events and LBW. Other significant associations with CS included a lack of maternal education, inadequate care of mother and fetus before birth, use of general anesthesia, younger maternal age, primiparity, early newborn death and disability, and the need for intensive care (NICU) for the newborn. Emergency procedures were associated with a higher rate of neonatal complications [15].

Vidovics et al. compared neonatal cord (arterial) blood pH, base excess, and the need for NICU care as a measure of neonatal outcomes in premature breech fetuses delivered via NVD or CS. They found that in newborns that had been delivered vaginally, the arterial pH was lower, while no statistically significant difference could be elicited for venous pH and the need for NICU care. This study concluded that routine CS for preterm breech fetuses cannot be recommended based on the mentioned findings [16]. Wylie et al. retrospectively studied over 2000 cases of VLBW deliveries and found that for preterm, VLBW but not SGA, and vertex-presenting fetuses, CS does not confer any added benefits in terms of neonatal survival [9].

Sentilhes et al. compared the outcomes for preterm twins, with the first in a cephalic presentation, who had been birthed via planned vaginal delivery and cesarean delivery. They defined adverse outcomes as bronchopulmonary dysplasia (BPD), IVH grade III or IV, necrotizing enterocolitis (NE), periventricular leukomalacia (PVL), and inpatient mortality. They found that NVD did not raise the likelihood of developing these severe complications or mortality [17]. Our study does show an increased risk of adverse outcomes secondary to CS though these findings are not statistically significant.

Kuper et al. conducted a study involving a retrospective cohort, which assessed the outcomes in singleton preterm births with respect to MOD and found no statistically significant difference [18]. This is in line with our findings as well. Ghi et al. studied preterm deliveries from 25 to 32 weeks and found that adverse fetal outcomes did not depend on MOD and CS carried risk for adverse maternal outcomes [19].

Holzer et al. found that whether CS would offer benefit depended upon the weight of the preterm fetus. According to them, SGA fetuses benefited from CS up until 31 weeks and appropriate-for-gestational-age (AGA) fetuses benefited till 28 weeks [20]. The advantages of CS thus depend upon fetal characteristics and these must be acknowledged during decision-making.

Blue et al. reported that CS performed before 30 weeks of gestation resulted in a greater risk of NRDS and admission to NICU in contrast to NVD before 30 weeks [21]. Of the negative outcomes studied by the current authors, respiratory distress was the only statistically significant adverse outcome of CS over NVD.

Despite adding value to the current literature, this study had certain limitations. It was hampered by a small sample size and the fact that it is a single-center study. A larger study with diversified sample size is required to ascertain the relationship between MOD and postnatal as well as neonatal outcomes.

## Conclusions

The present study concludes that there is no difference in neonatal outcomes in terms of IVH, seizures, SDH, and APGAR score of <7. However, respiratory distress was significantly more common in babies of women who delivered via CS. Further large-scale studies are required to ascertain the association between fetal outcomes in women undergoing CS versus those who have spontaneous vaginal delivery.
